# Supplementary figures and images for: Computational prediction of the pathogenic variants of arachidonate 5-lipoxygenase activating protein using Molecular Dynamics simulation
Source: PLoS One. 2025 Jul 30;20(7):e0329126. doi: 10.1371/journal.pone.0329126 (PMC12310011; doi:10.1371/journal.pone.0329126)

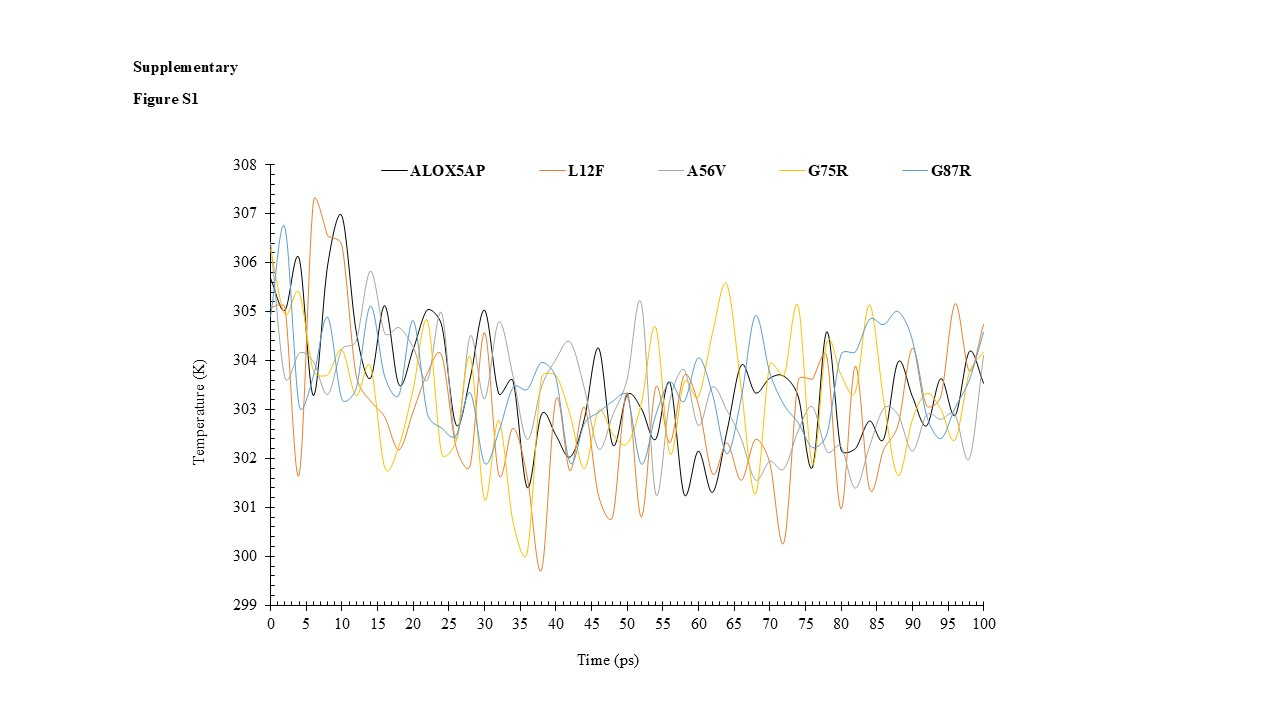

Supplement: S1 Fig — (TIF) [file pone.0329126.s001.tif]

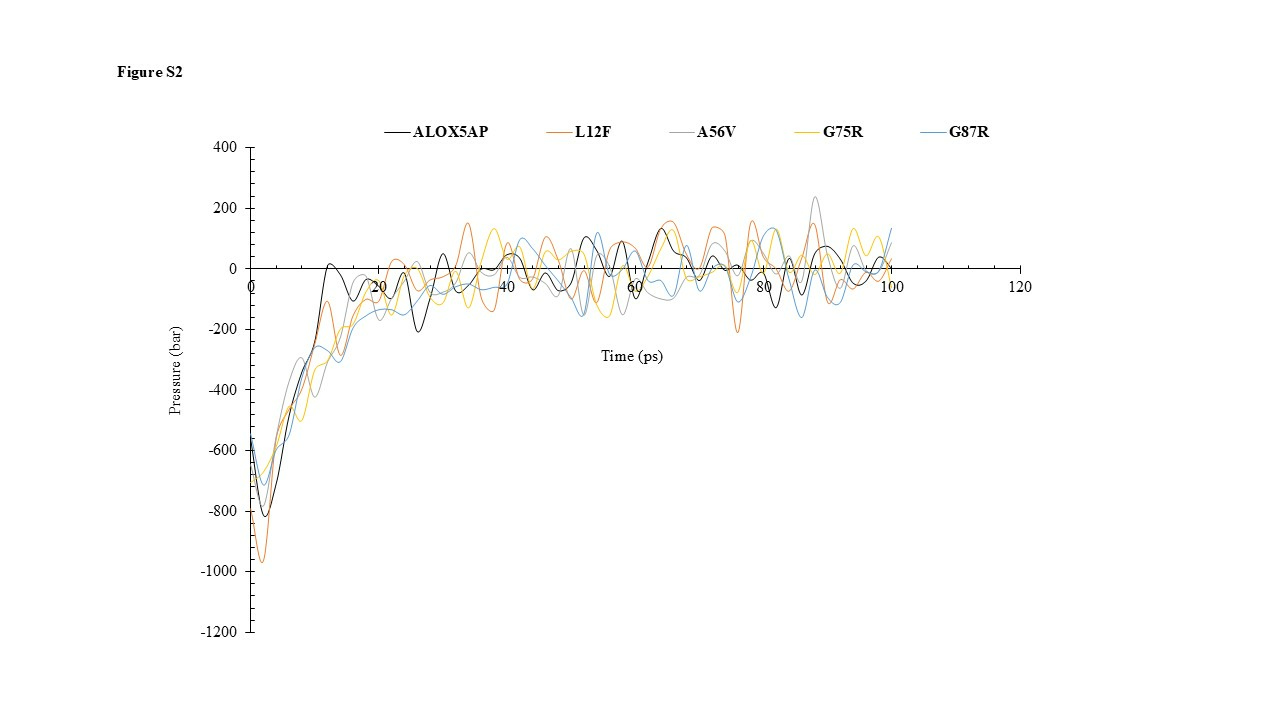

Supplement: S2 Fig — (TIF) [file pone.0329126.s002.tif]

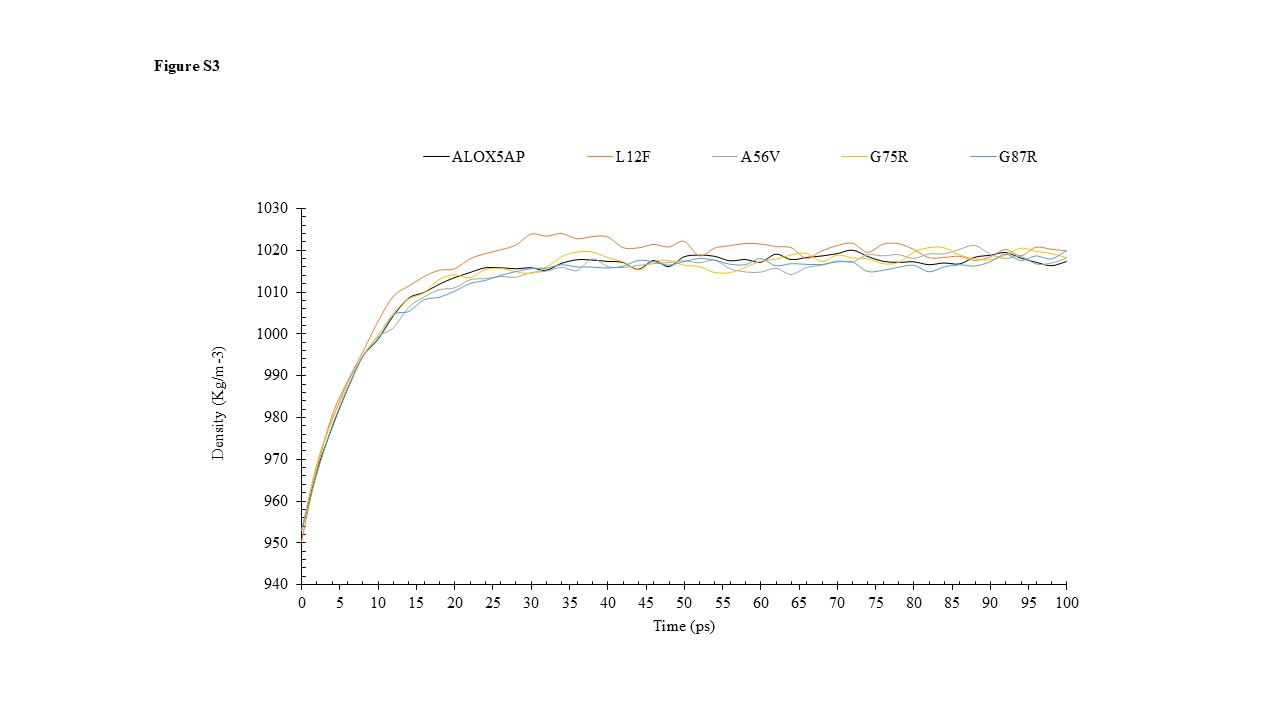

Supplement: S3 Fig — (TIF) [file pone.0329126.s003.tif]
