# Supplementary material for: Computational prediction of the pathogenic variants of arachidonate 5-lipoxygenase activating protein using Molecular Dynamics simulation
Source: PLoS One. 2025 Jul 30;20(7):e0329126. doi: 10.1371/journal.pone.0329126 (PMC12310011; doi:10.1371/journal.pone.0329126)
Supplement: Attachment — Submitted filename: PONE-D-25-17796.docx [file pone.0329126.s004.docx]

The manuscript contains numerous grammatical errors and typographical mistakes throughout. A thorough revision is necessary before resubmission. Editing by a native English speaker or professional language editing service is strongly recommended.

**Section 2.2: Data Collection**

- References are missing. Please provide appropriate citations to support the methodology described.

**Section 2.5: Molecular Dynamics (MD) Simulations Using GROMACS**

- The section lacks sufficient citations. Key methodological steps and tools used in the simulations should be properly referenced.

**Section 3.1: Prediction of Deleterious nsSNPs in ALOX5AP**

- The classification of mutations is not adequately referenced. For example, the manuscript states that SIFT classified all examined nsSNPs as harmful, with scores between 0 and 0.03. However, the rationale for selecting this threshold is not provided or supported with references.
- Throughout this section, several predictive tool results (e.g., SNPs&GO, PROVEAN, PhD-SNPg) are reported without proper citation or explanation of the basis of score ranges used for classification. This reduces the credibility of the findings and should be addressed.

**Discussion**

- The discussion lacks scientific depth and critical comparison with findings from other published studies.
- The opening part of the discussion reads more like an introduction and should be revised to focus on interpreting results.
- The impact of the identified mutations, particularly in relation to cardiovascular diseases, needs to be discussed in more detail. For example, although the figures suggest that the L12F variant causes significant structural compaction of ALOX5AP, the manuscript fails to explain how this structural change could affect protein function or contribute to cardiovascular disease mechanisms.
- The sentence: *“Our result in inconsistent with a study reported that the p.L12F variant has no detrimental effect on the pancreatic secretory trypsin inhibitor protein(47), this inconsistency may because of the different protein examined in that study(47).”* is problematic both in grammar and logic. If the compared study involves an entirely different protein, then drawing a direct comparison based on the same amino acid substitution (L12F) is inappropriate due to differences in tertiary structure and protein context. This kind of comparison is premature and scientifically unsound.

**Conclusion**

- The conclusion section would benefit from a brief discussion of the study’s limitations. Additionally, future research directions should be clearly outlined to enhance the manuscript’s overall impact.
