# Supplementary material for: Computational prediction of the pathogenic variants of arachidonate 5-lipoxygenase activating protein using Molecular Dynamics simulation
Source: PLoS One. 2025 Jul 30;20(7):e0329126. doi: 10.1371/journal.pone.0329126 (PMC12310011; doi:10.1371/journal.pone.0329126)
Supplement: Attachment — Submitted filename: Response to Reviewers.docx [file pone.0329126.s005.docx]

Dear Editor, Dear reviewers

Thank you very much for your consideration. We also thank you and the reviewers for the questions and the comments that significantly improved our manuscript. Please find below step-by-step response to all questions and comments.

Best regards,

Imadeldin Elfaki, PhD

The manuscript contains numerous grammatical errors and typographical mistakes throughout. A thorough revision is necessary before resubmission. Editing by a native English speaker or professional language editing service is strongly recommended.

Authors

Thank you very much. We are very sorry that manuscript contains numerous grammatical errors and typographical mistakes. English is revised carefully.

**Section 2.2: Data Collection**

- References are missing. Please provide appropriate citations to support the methodology described.

Authors

- Thank you very much. Appropriate citations to support the methodology described are provided. Please see ref 24 to 45.

**Section 2.5: Molecular Dynamics (MD) Simulations Using GROMACS**

- The section lacks sufficient citations. Key methodological steps and tools used in the simulations should be properly referenced.
- Authors
- Thank you very much. In molecular dynamics (MD) simulation the temperature, pressure, and density [1, 2] characteristics of the wild-type ALOX5AP protein and its mutants (L12F, A56V, G75R, and G87R) were examined throughout a 100 ps simulation period to evaluate the structural and dynamic impacts of these variants. to examine the structural stability and flexibility of ALOX5AP key metrics such as the root-mean-square deviation (RMSD) [1, 2], which assesses the ALOX5AP overall structural stability, and root-mean-square fluctuation (RMSF) [1, 2], which evaluated the flexibility of individual amino acid residue. We also examine the radius of gyration (Rg) which gives an understanding of the overall dimensions of ALOX5AP, and solvent- accessible surface area (SASA) that is a bio-molecular surface area accessibility to solvent molecules [1, 3]. Hydrogen bonds analysis was also examined, HB is an important factor in protein stability, structural integrity, and interactions[4]. To enhance the data interpretation, custom Python scripts were developed [5] to generate visualizations of these parameters, enabling a comprehensive understanding of the simulation results.

**Section 3.1: Prediction of Deleterious nsSNPs in ALOX5AP**

- The classification of mutations is not adequately referenced. For example, the manuscript states that SIFT classified all examined nsSNPs as harmful, with scores between 0 and 0.03. However, the rationale for selecting this threshold is not provided or supported with references.
- Authors
- Thank you very much. In SIFT Score, the amino acid substitution is predicted damaging if the score is <= 0.05[6], please also see website <https://sift.bii.a-star.edu.sg/www/SIFT_help.html>. The SIFT Score of the selected variants L12F, A56V, G75R and G87R variants was 0 (Table1). This score is predicted damaging or deleterious to the protein.

- Throughout this section, several predictive tool results (e.g., SNPs&GO, PROVEAN, PhD-SNPg) are reported without proper citation or explanation of the basis of score ranges used for classification. This reduces the credibility of the findings and should be addressed.
- Authors
- -Thank you very much. In the SNPs&Go, score of 0.5 is selected to distinguish between benign (*t*≤0.5) and pathogenic (*t*>0.5) SNP[7]. SNPs&GO categorized all four examined nsSNPs as "pathogenic," with pathogenicity probability (Path_Prop) between 0.584 and 0.953[7] (Table2).
- -We did not employ PROVEAN in this study.
- - The PhD-SNP classified all four variations as "disease-associated," with reliability indices (RI) between 2 and 9, indicating the confidence in the predictions[8].

**Discussion**

- The discussion lacks scientific depth and critical comparison with findings from other published studies.
- Authors
- Thank you very much. A comparison with findings from other published studies was added in a separated paragraph. Changes are highlighted.

- The opening part of the discussion reads more like an introduction and should be revised to focus on interpreting results.

Authors

opening part of the is revised and focused on interpreting results

- The impact of the identified mutations, particularly in relation to cardiovascular diseases, needs to be discussed in more detail. For example, although the figures suggest that the L12F variant causes significant structural compaction of ALOX5AP, the manuscript fails to explain how this structural change could affect protein function or contribute to cardiovascular disease mechanisms.
- Authors
- Thank you very much. We explained how this structural change due to the mutation could affect ALOX5AP function or contribute to cardiovascular disease mechanisms.

- The sentence: *“Our result in inconsistent with a study reported that the p.L12F variant has no detrimental effect on the pancreatic secretory trypsin inhibitor protein(47), this inconsistency may because of the different protein examined in that study(47).”* is problematic both in grammar and logic. If the compared study involves an entirely different protein, then drawing a direct comparison based on the same amino acid substitution (L12F) is inappropriate due to differences in tertiary structure and protein context. This kind of comparison is premature and scientifically unsound.
- authors
- Thank you very much. We remove the sentence “Our result in inconsistent with a study reported that the p.L12F variant has no detrimental effect on the pancreatic secretory trypsin inhibitor protein.

**Conclusion**

- The conclusion section would benefit from a brief discussion of the study’s limitations. Additionally, future research directions should be clearly outlined to enhance the manuscript’s overall impact.
- Authors
- Thank you very much. Some study limitations and suggested future research direction are added to the conclusion.

REFERENCES

1.	Hasnain MJU, Shoaib M, Qadri S, Afzal B, Anwar T, Abbas SH, Sarwar A, Talha Malik HM, Tariq Pervez M: **Computational analysis of functional single nucleotide polymorphisms associated with SLC26A4 gene**. *PLoS One* 2020, **15**(1):e0225368.

2.	Alomair L, Mustafa S, Jafri MS, Alharbi W, Aljouie A, Almsned F, Alawad M, Bokhari YA, Rashid M: **Molecular Dynamics Simulations to Decipher the Role of Phosphorylation of SARS-CoV-2 Nonstructural Proteins (nsps) in Viral Replication**. *Viruses* 2022, **14**(11).

3.	Rani N, Boora N, Rani R, Kumar V, Ahalawat N: **Molecular dynamics simulation of RAC1 protein and its de novo variants related to developmental disorders**. *J Biomol Struct Dyn* 2024, **42**(24):13437-13446.

4.	Zhang D, Lazim R: **Application of conventional molecular dynamics simulation in evaluating the stability of apomyoglobin in urea solution**. *Sci Rep* 2017, **7**:44651.

5.	Irrgang ME, Davis C, Kasson PM: **gmxapi: A GROMACS-native Python interface for molecular dynamics with ensemble and plugin support**. *PLoS Comput Biol* 2022, **18**(2):e1009835.

6.	Ng PC, Henikoff S: **Predicting deleterious amino acid substitutions**. *Genome Res* 2001, **11**(5):863-874.

7.	Capriotti E, Martelli PL, Fariselli P, Casadio R: **Blind prediction of deleterious amino acid variations with SNPs&GO**. *Hum Mutat* 2017, **38**(9):1064-1071.

8.	Capriotti E, Calabrese R, Casadio R: **Predicting the insurgence of human genetic diseases associated to single point protein mutations with support vector machines and evolutionary information**. *Bioinformatics* 2006, **22**(22):2729-2734.
