# Supplementary material for: Computational prediction of the pathogenic variants of arachidonate 5-lipoxygenase activating protein using Molecular Dynamics simulation
Source: PLoS One. 2025 Jul 30;20(7):e0329126. doi: 10.1371/journal.pone.0329126 (PMC12310011; doi:10.1371/journal.pone.0329126)
Supplement: Attachment — Submitted filename: response to reviewers 7 7 25 afternoon.docx [file pone.0329126.s006.docx]

Dear Editor

Thank you very much for the considering our paper. We also thank you and the reviewers for the comments and questions that significantly improved our manuscript. Please find below responses to questions and comments. Changes are highlighted.

Best regards,

Imadeldin Elfaki, PhD

1) **Drop the word "(MD)" from the title.**

Authors

Thank you very much. MD is dropped from the title.

2) **Section 2.1 heading, check the spelling of "Plane".**

Authors

Thank you very much: The spelling of ‘’plane’’ is corrected.

3) **A large number of abbreviations have been used. Compile a list of abbreviations used and place it before the References section**

Authors

Thank you very much. A list of abbreviations used is prepared and placed before the References section

4) **There spacing issues and other typos throughout the manuscript. For instance, there is no space between Figure and figure numbers. Get your manuscript thoroughly proofread.**

Authors

Thank you very much. Typos are corrected; the manuscript is thoroughly proofread.
